# Enhancing the monitoring of fallen stock at different hierarchical administrative levels: an illustration on dairy cattle from regions with distinct husbandry, demographical and climate traits

**DOI:** 10.1186/s12917-020-02312-8

**Published:** 2020-04-14

**Authors:** Amanda Fernández-Fontelo, Pedro Puig, German Caceres, Luis Romero, Crawford Revie, Javier Sanchez, Fernanda C. Dorea, Ana Alba-Casals

**Affiliations:** 1 Chair of Statistics, School of Business and Economics, Humboldt Universität zu Berlin, Berlin, Germany; 2 Departament de Matemàtiques, Universitat Autònoma de Barcelona, Cerdanyola del Vallès, Barcelona, Spain; 3 Subdirección General de Sanidad e Higiene Animal y Trazabilidad. Ministerio de Agricultura y Pesca, Alimentación (MAPA), Madrid, Spain; 4 Centre for Veterinary Epidemiological Research, AVC, University Prince Edward Island (UPEI), Charlottetown, Canada; 5 Department of Computer and Information Sciences, University of Strathclyde, Glasgow, Scotland, UK; 6 Department of Disease Control and Epidemiology, National Veterinary Institute (SVA), Uppsala, Sweden; 7 Department of Veterinary Population Medicine, College of Veterinary Medicine, University of Minnesota, St. Paul, USA; 8 Centre de Recerca en Sanitat Animal (CReSA), Institut de Recerca i Tecnologia Agroalimentàries (IRTA), Cerdanyola del Vallàs, Barcelona, Spain

**Keywords:** Fallen stock, Dairy cattle, Syndromic surveillance, Hierarchical time series, ARIMA models, Spain

## Abstract

**Background:**

The automated collection of non-specific data from livestock, combined with techniques for data mining and time series analyses, facilitates the development of animal health syndromic surveillance (AHSyS). An example of AHSyS approach relates to the monitoring of bovine fallen stock. In order to enhance part of the machinery of a complete syndromic surveillance system, the present work developed a novel approach for modelling in near real time multiple mortality patterns at different hierarchical administrative levels. To illustrate its functionality, this system was applied to mortality data in dairy cattle collected across two Spanish regions with distinct demographical, husbandry, and climate conditions.

**Results:**

The process analyzed the patterns of weekly counts of fallen dairy cattle at different hierarchical administrative levels across two regions between Jan-2006 and Dec-2013 and predicted their respective expected counts between Jan-2014 and Jun- 2015. By comparing predicted to observed data, those counts of fallen dairy cattle that exceeded the upper limits of a conventional 95% predicted interval were identified as mortality peaks. This work proposes a dynamic system that combines hierarchical time series and autoregressive integrated moving average models (ARIMA). These ARIMA models also include trend and seasonality for describing profiles of weekly mortality and detecting aberrations at the region, province, and county levels (spatial aggregations). Software that fitted the model parameters was built using the R statistical packages.

**Conclusions:**

The work builds a novel tool to monitor fallen stock data for different geographical aggregations and can serve as a means of generating early warning signals of a health problem. This approach can be adapted to other types of animal health data that share similar hierarchical structures.

## Background

Animal health surveillance is essential for planning and implementing efficient prevention and control measures that are important for animal and public health [1, 2]. Current advances in data mining techniques and spatial-temporal analysis facilitate the development of novel animal health syndromic surveillance (AHSyS) approaches. These methods allow us to obtain information regarding the health status of animal populations to be extracted from diverse automated data sources of a non-specific nature in near real-time [3–5]. AHSyS may enhance traditional animal surveillance systems by identifying subpopulations at high risk, assessing the impact of previous prevention or control measures, supporting claims of freedom from disease, and serving as an early warning system [6]. This AHSyS approach involves the continuous analysis of mortality data registered at farm level. The potential of mortality data for this purpose has been demonstrated in previous studies conducted in a number of European countries [7–12]. In Catalonia (North-Eastern Spain), patterns of fallen bovine were modelled, for the main cattle production types between 2006 and 2013, using time series analyses [7, 8]. In those studies, bovine fallen stock data weekly aggregated at the regional level were fitted using autoregressive integrated and moving average (ARIMA) models. These models included trend and seasonal patterns as covariates. According to these ARIMA models and assuming regularity in the bovine population patterns over time, both the number of visits to carcass disposal and the total weight collected in these visits (in kg) were predicted at n-week ahead. These models provided information to assess the impacts of different events occurred over time at the regional level. However, to identify high-risk subpopulations and/or to serve as an early warning system, the number of fallen bovines must be assessed at more spatially discrete administrative aggregations. With this aim, fallen bovine data aggregated at province and county levels were plotted using hierarchical time series structures (HTS) according to the methodology proposed by Hyndman et al. [13, 14]. These plots indicated that patterns in fallen bovine data varied substantially among different subpopulations. This implied that multiple time series would have to be modelled at more refined spatial scales in order to get well-fitting models for different spatial aggregations.

This work is a methodological extension to the modelling approach introduced in the previous papers of Alba et al. [7, 8]. In [7, 8] the indicator of fallen stock was only modelled at the region level. Although the modelling of these data aggregated at higher levels allowed the quantification of the total impact (or lack thereof) on a population in the event of a potential animal threat, using only this level of analysis, the likelihood of detecting local and moderate variations was limited. The problem was that the selection of an appropriate and parsimonious ARIMA model accounting for seasonality and trend for specific time series involved the testing and checking of many combinations of values for each parameter. This process was not automatic and modelling many time series from different subpopulations was very tedious. Previously, Alba et al. plotted the time series of smaller subpopulations using HTS structures. However, these HTS did not quantify temporal patterns, forecast mortality based on previous observations, or detect aberrations over time at different administrative levels. The present study solves this problem by creating an automated process to analyze patterns of multiple fallen cattle subpopulations with lower numbers and high variability. This modelling process aimed to analyze and predict fallen stock patterns at both low and high geographical levels. Moreover, in this work, we used as an indicator of fallen dairy cattle the “counts of fallen bovines”, which is more accurate than the “counts of carcass disposal visits” or the “total of kg collected” used before by Alba et al. 2015 [7].

To illustrate the functionality of our method in different contexts, it was put in motion by using the retrospective fallen stock data of dairy cattle collected between 2006 and 2015 in two regions of Spain with different demographical structure, husbandry systems, and climate conditions.

## Results

These results describe the basic traits of two Spanish regions with distinct dairy cattle populations (R1 and R2) and the monitoring of counts of fallen bovines collected by week at three administrative levels (i.e., region, province, and county) over a 10-year period.

**Fig. 1:**
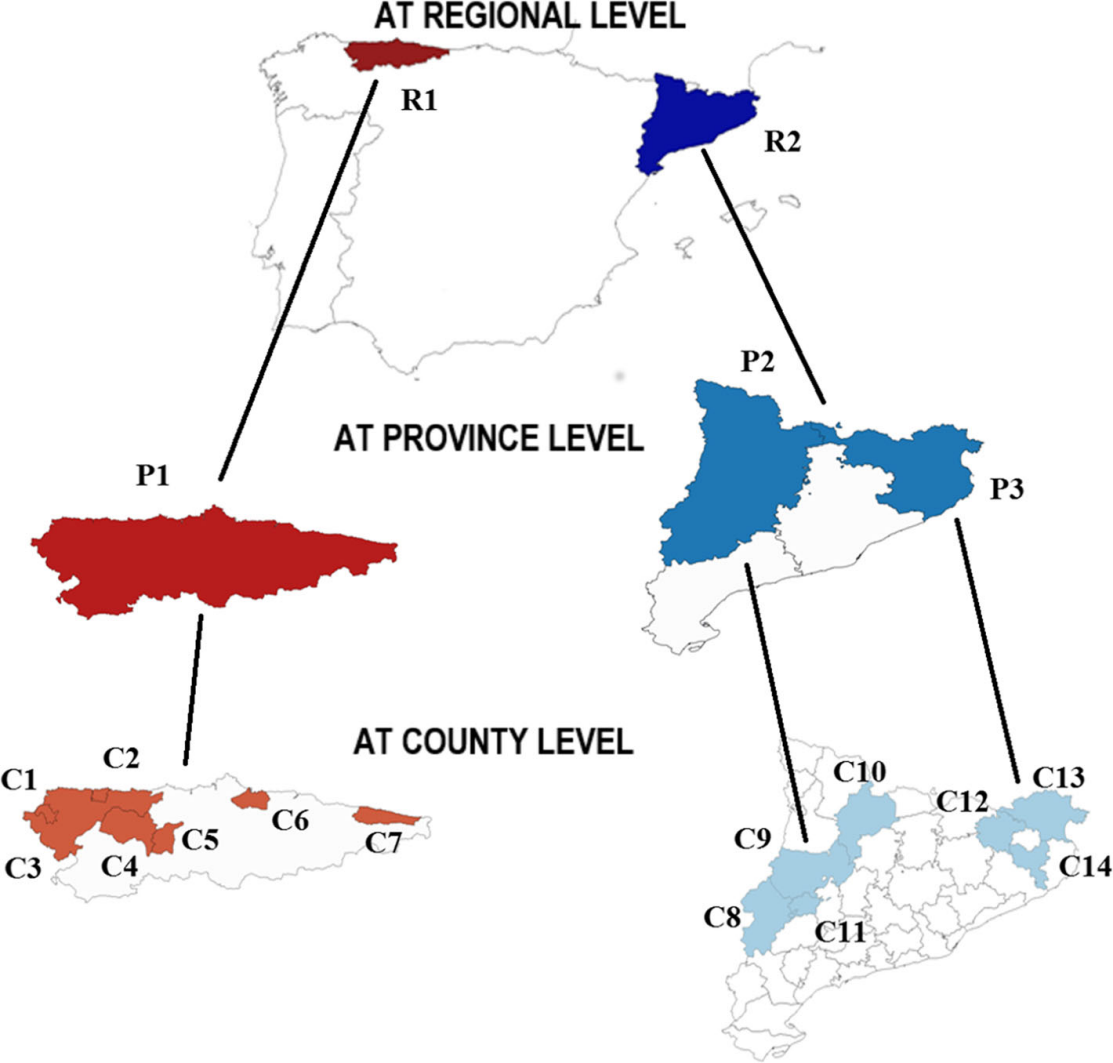
Spanish regions, provinces, and counties within which the levels of fallen dairy cattle were monitored. The figure is own-created

The region R1 had registered 3469 dairy farms (with an annual median of 2958 dairy farms and 117,572 heads of cattle), while region R2 had 984 farms registered (with an annual median of 797 farms and 124,953 heads of cattle). This system covered 77 and 81% of all these dairy farms of R1 and R2, respectively. Figure 1 shows the administrative levels monitored in this study, i.e. across R1 seven of its 17 counties, and across R2 two of its four provinces, and in seven of its 26 counties.

Over this period of study in these two regions, the number of dairy cattle farms decreased and the sizes of the herds increased; but the total number of bovines in these populations did not change substantially. R1 had 3.4 times more dairy farms than R2 and the herd size was 2.8 times smaller. This difference was also evidenced consistently at smaller administrative levels, such as provinces and counties. The proportion of herds in extensive production also varied, being around 0% in R2 and 25% in R1. Regarding the climate, this was quite similar across counties of R1, but differed among the counties of R2. Moreover, in some counties of R2 the high proportion of herds housed all-year-round combined with a continental climate with warm summers could cause heat stress problems and an increase of mortality. Initially, each administrative level was described through the number of dairy farms, the herd size, the type of husbandry, the climate, and the number of fallen bovines collected in total and by week. This preliminary exploration indicated that most of these subpopulations differed substantially in relation to demographical and climate traits as shown in Table 1.

**Table 1:** Description of dairy cattle populations under monitoring with their respective patterns of mortality and mortality aberrations detected at each level

Zones of study	Demographical and climate traits of populations monitored			Carcasses collected		Patterns of fallen bovines by week (Jan 2006- Dec 2013)				Mortality Aberrations (Jan 2014-Jun 2015)	
	Nr farms (Median)	Farm size Median (min-max)	Climate	Total nr	By week Median (min-max)	ARIMA (p, d, q)	Trend	Seasonal effect (Warm) (% of increase and period)	Seasonality effect (Cold) (% of increase and period)	Nr of peaks	Nr of affected farms Median (min-max)
**R1(=P1)**	**2681**	**74 (1–561)**	**Oceanic coastal**	**109,744**	**221 (151–326)**	**1,0,1**	**+ 0.086**	**8% Jun-Aug**	**22% Oct-Feb**	**4**	**145 (105–184)**
**C1**	**343**	**77 (1–369)**	**Oceanic coastal**	**16,114**	**32 (13–61)**	**0,1,1**	**+ 0.007**	**14% May-Aug**	**26%Oct-Feb**	**6**	**35 (30–44)**
**C2**	**302**	**71 (1–407)**	**Oceanic coastal**	**15,440**	**31 (10–58)**	**0,1,1**	**_**	**9% May-Aug**	**17%Oct-Feb**	**4**	**28 (13–37)**
**C3**	**400**	**63 (1–561)**	**Oceanic coastal**	**15,145**	**30 (14–58)**	**1,0,1**	**+ 0.009**	**16% May- Aug**	**16%Oct -Feb**	**6**	**29 (3–35)**
**C4**	**425**	**75 (1–430)**	**Oceanic coastal**	**17,205**	**34 (12–68)**	**0,1,1**	**+ 0.022**	**8% May-Aug**	**11%Oct - Feb**	**2**	**48 (44–51)**
**C5**	**143**	**84 (2–240)**	**Oceanic coastal**	**5924**	**12 (3–25)**	**1,0,1**	**+ 0.004**	**_**	**15%Oct-Jan**	**4**	**15 (13–18)**
**C6**	**314**	**82 (1–487)**	**Oceanic coastal**	**14,850**	**29 (13–62)**	**0,1,1**	**+ 0.125**	**22% May-Aug**	**35%Oct-Jan**	**1**	**30**
**C7**	**71**	**88 (5–218)**	**Oceanic coastal**	**4350**	**8 (0–20)**	**1,0,1**	**−0.006**	**_**	**15%Oct-Mar**	**2**	**10 (9–11)**
**R2**	**799**	**198 (1–3639)**	**Diverse climates: Mediterranean and Alpine**	**153,520**	**308 (144–502)**	**0,1,1**	**+ 0.229**	**61% May-Aug**	**85%Oct-Jan**	**1**	**38**
**P2**	**212**	**220 (1–3639)**	**Mediterranean continental**	**49,557**	**104 (40–197)**	**0,1,1**	**+ 0.110**	**96% May-Aug**	**138%Oct-Jan**	**1**	**49**
**C8**	**22**	**297 (3–3369)**	**Mediterranean continental warm summers**	**9331**	**17(2–52)**	**0,1,1**	**+ 0.037**	**145% May- Set**	**184% Oct- Jan**	**7**	**5 (2–9)**
**C9**	**21**	**526 (14–2005)**	**Mediterranean continental warm summers**	**10,309**	**19 (3–55)**	**2,1,2**	**+ 0.031**	**145% May- Set**	**164%Oct- Jan**	**4**	**7 (1–7)**
**C10**	**98**	**192 (1–1556)**	**Mediterranean continental subhumid**	**18,107**	**36 (9–75)**	**0,1,1**	**+ 0.040**	**83% May-Aug**	**125%Oct- Jan**	**1**	**28**
**C11**	**25**	**206 (17–1403)**	**Mediterranean continental warm summers**	**9055**	**17(4–58)**	**4,1,2**	**+ 0.012**	**100% May-Set**	**134%Oct-Jan**	**1**	**5**
**P3**	**308**	**191 (6–1933)**	**Mediterranean Continental subhumid /costal**	**56,274**	**106 (54–200)**	**1,0,1**	**+ 0.079**	**59% May-Aug**	**78%Oct- Jan**	**7**	**5 (2–9)**
**C12**	**41**	**197 (6–559)**	**Mediterranean continental subhumid**	**7701**	**15 (2–37)**	**1,0,1**	**+ 0.009**	**72% May-Aug**	**111% Oct-Jan**	**1**	**11**
**C13**	**61**	**231 (7–905)**	**Mediterranean costal**	**14,265**	**28 (11–73)**	**0,1,1**	**+ 0.021**	**67% May- Aug**	**72%Oct- Jan**	**3**	**9 (8–15)**
**C14**	**54**	**228 (6–1933)**	**Mediterranean costal**	**14,778**	**29 (10–70)**	**3,1,1**	**+ 0.011**	**41% May-Aug**	**72%Set-Jan**	**2**	**9 (7–10)**

Zones of study: *R* Region, *C* County, *P* Province

ARIMA(pdq) where p = order of autocorrelation, d = differentiation, q = order moving average

Plots of the HTS of weekly counts of fallen bovines at region, province and county levels are shown in S2 and S3 Figs.

The respective plots of HTS for each region indicated that the fallen stock patterns varied among subpopulations. For this reason, it was important to model these patterns at each administrative level to obtain robust information, detect and investigate abnormal mortality events and support the decision-making.

For each administrative aggregation the most appropriate ARIMA model, which could include trend and seasonal effects, was fitted based on the training dataset collected between Jan-2006 and Dec-2013.

The main traits of the two dairy cattle subpopulations and the monitored fallen stock data are summarized in Table 1.

**Fig. 2:**
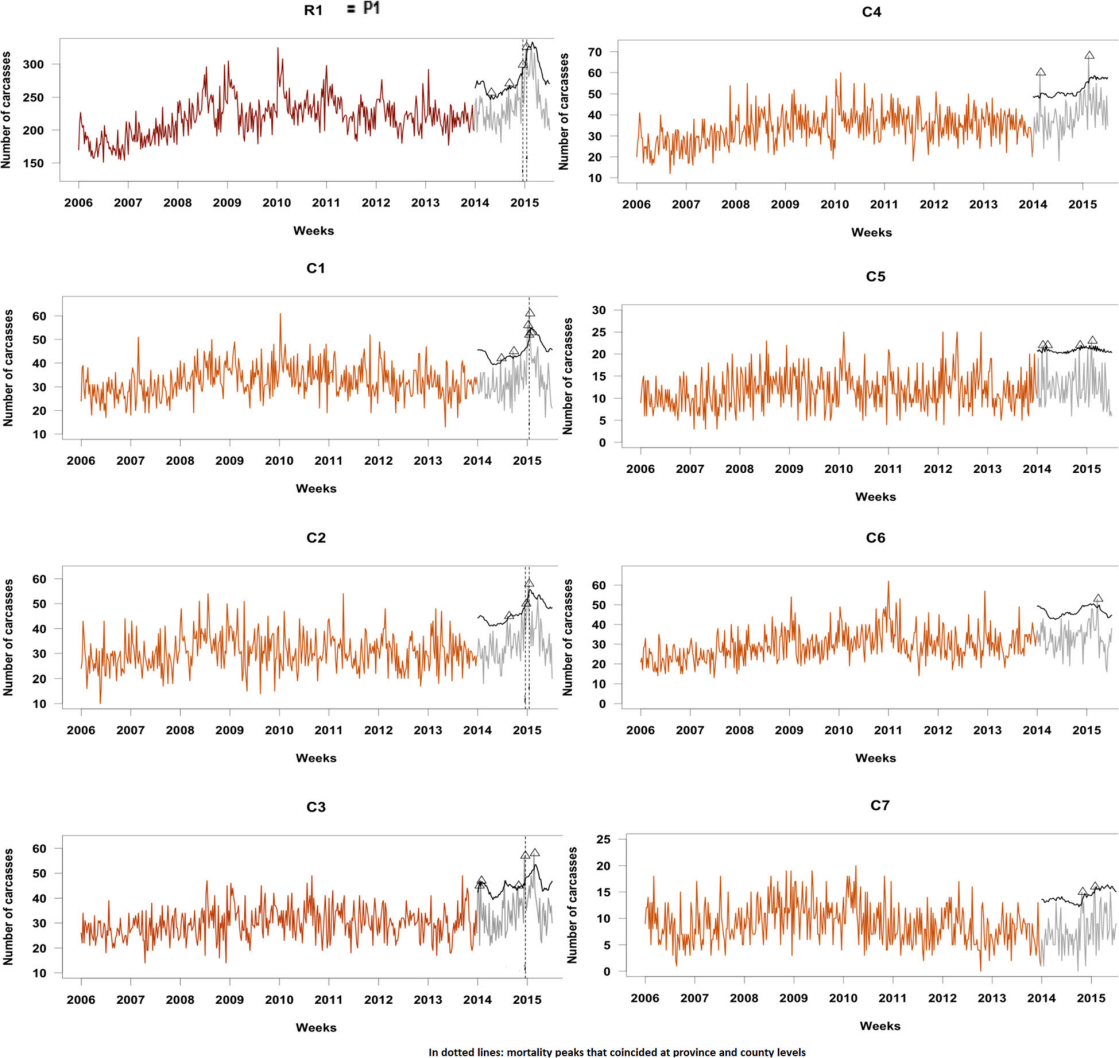
Time series plots of fallen bovines in the training data set collected weekly together with mortality peaks **(A)** detected in the testing dataset for R1 (P1)

**Fig. 3:**
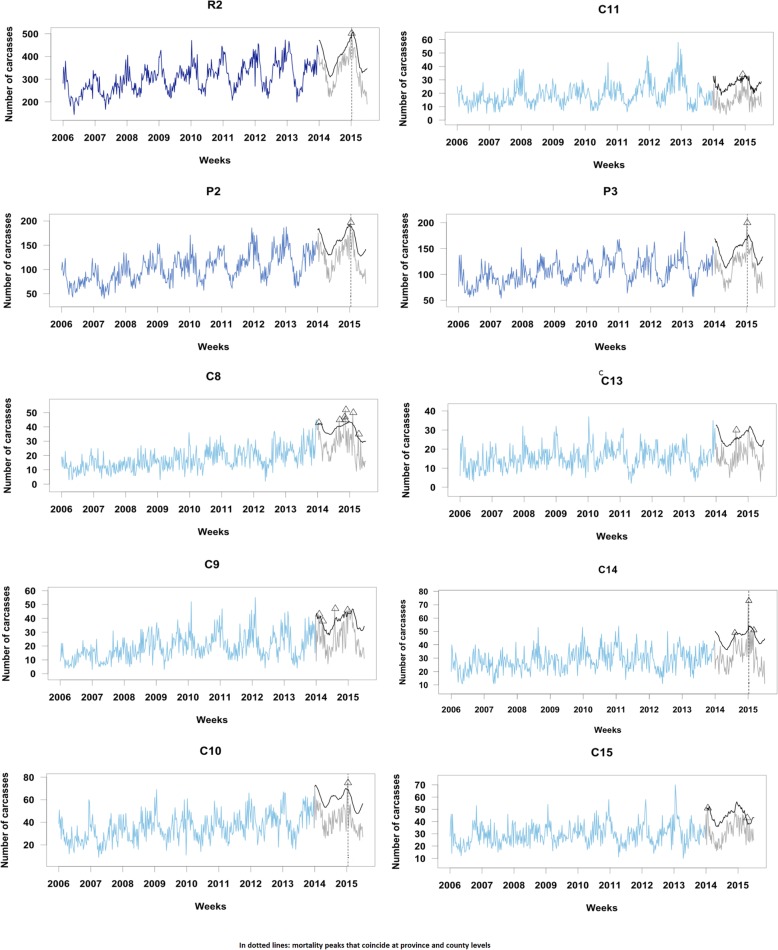
Time series plots of fallen bovines in the training dataset collected by week together with mortality peaks **(A)** detected in the testing dataset for R2

In most of these administrative levels of both regions R1 and R2, the fallen dairy cattle presented annual and biannual seasonality, with an increasing trend over time. Interestingly, this pattern was consistent for all the counties of R2, but differed among counties of R1. Both regions (i.e., all the series in R2 and six series of R1) exhibited an increased trend and seasonal pattern that rose during January and February, coinciding with the coldest season (Figs. 2 and 3). During July and August, in the warmest period of the year, there was also an increase of mortality, but this rise was only very evident in R2 and especially in some counties (see Table 1). The weekly counts of fallen bovines collected were much lower in R1 than in R2 (i.e., a median of 221 heads in R1, versus 308 in R2). However, the number of farms involved in each peak was higher in R1 than in R2 (a median of 145 farms in R1, versus 38 in R2). Moreover, it should be noted that in R1 the trend and seasonal patterns among counties were more disparate and irregular, mainly in counties with lower counts such as C5 and C7. Finally, based on these models, the fallen bovines expected by week were forecasted for each subpopulation during Jan-2014 and Jun-2015. Those observations that exceeded the predicted upper confidence limits were signaled as mortality aberrations.

Considering a conventional upper 95% confidence limit, six mortality aberrations would be signaled at a province level (four in R1 and two in R2) affecting a total of 553 farms (466 in R1 and 87 in R2). Two of the four peaks detected in R1 at a province level were also detected at a county level, while in R2 all peaks detected at a province level would be also detected at a county level (see Figs. 2 and 3).

In both regions there was a marked increase in counts of mortality during Jan-2015, being identified as unusual events in all administrative aggregations except for one county (C14 in R2).

## Discussion

Despite the growing interest of using syndromic information for AHSyS [6], its implementation as a part of an early warning system has to be validated in the field yet. In many European countries, bovine fallen stock data have been gathered consistently over several years by carcass disposal services, covering a significant proportion of active farms. The potential use of bovine fallen stock data for AHSyS was described in previous studies [7–12]. In [9, 11], some authors also demonstrated the usefulness of these methods for assessing the impact of past events, such as the incursion of bluetongue or the heat waves in 2003 and 2006 in the French cattle population. However, many technical challenges must still be faced when putting in place the monitoring of bovine fallen stock as part of an alert system or as a support tool for decision making [7, 10].

One of the main challenges relates to the heterogeneity of the animal populations under surveillance. In the context of animal health surveillance, the target population usually comprises different subpopulations with disparate managements, structures and risks. Moreover, the official animal health services of different administrative levels coordinate which prevention and control measures should be applied in each population. The modelling approach proposed in this study allows for an assessment of bovine fallen stock baselines and the detection of mortality peaks in different populations. The results of this study indicate that the baselines of fallen bovines vary among different administrative aggregations. Consequently, an effective monitoring system based on fallen stock data should be able to provide information and detect alerts for each population in near real time.

In this work we combined the expertise from veterinary epidemiologists and statisticians to lead to a robust tool that constitutes an important step towards its implementation. Its functionality has been illustrated across two regions of Spain from populations with different husbandry systems and environmental conditions. Accordingly, this study demonstrated the capability of this approach to analyze, model and predict, in an automatic way, the number of fallen dairy cattle by week for different subpopulations.

The system used as an indicator of mortality the number of carcasses collected each week, assuming that the studied populations remained relatively constant over time. To support this assumption, the behavior of the dairy population was explored year-by-year for both regions. This exploration indicated that, although the number of dairy cattle farms decreased throughout the study, the number of dairy cattle heads slightly increased.

Initially, the weekly counts of fallen bovines were described and plotted for all administrative levels using HTS. This technique was used to explore, compare fallen dairy cattle patterns among different administrative levels, and select those series that would be immediately modelled using a time series methodology such ARIMA modelling, including trend and seasonality as covariates.

A computer programming routine was developed to fit an ARIMA model for each selected administrative aggregation, forecast the counts of fallen dairy cattle that would be expected at n-ahead period and identify those counts that exceeded the upper 95% confidence limit, as presumed mortality peaks. This system can be applied to different dairy cattle population structures (such as those seen in R1 or R2).

Classical time series models such ARIMA had the advantage that they can deal with trend and seasonal components as well as temporal correlation structures, which in the case of fallen bovine data were especially marked (see S1 and S2 Tables). The trend and seasonal components could be modelled using trigonometric functions [15], simple exponential smoothing [16, 17] or Holt-Winters [18, 19]. The temporal autocorrelation could be fitted using autoregressive integrated moving average (ARIMA) models [20, 21]. In this work ARIMA modelling, including possible trends and seasonal effects as covariates, demonstrated to best fit data related to weekly counts of fallen bovines without requiring excessive computation time and using standard software. Moreover, if the response variable would depart from the normality assumption (e.g. time series with counts less than 10 in average), these models could be replaced by Integer-valued Autoregressive (INAR) models [12, 22]. Other more sophisticated methodologies of time series have been proposed. For instance, Le Strat and Carrat [23] used Hidden Markov Models (HMM) to monitor epidemiological data by segmenting series into epidemic and non-epidemic unobserved phases. These unobserved phases could be modelled by a two-state homogeneous Markov chain of order 1 (epidemic and non-epidemic); higher orders could also be considered. As an alternative, Martinez-Beneito [24] proposed a Markov switching model in which the response variable did not depend only on the hidden states, but also on the lagged observable variable. However, according to [25], these methodologies both require extensive computation time and are therefore not suited to monitoring epidemiological data in near real time. Furthermore, in general, Bayesian methods were computationally intensive and strongly dependent on prior information, which in our case did not exist [26]. Parametric regression techniques, including a variety of models such as simple regression, could be explored. These include naive regression that estimated the response variable at time by the mean of responses at times t-1, t and t + 1 to adjust for possible seasonal effects [27] or extended regression that incorporated trigonometric functions with linear trend accounting for both trends and seasonal effects [28]. Additionally, when the response variable departed from normality *(*i.e. low counts with many zeros), some authors [29–31] have proposed Poisson regression models with logarithmic link to model possible trends and seasonal patterns. Hierarchical generalized linear mixed models [32] are another methodology for fitting non-normal correlated data. Another approach of semiparametric regression could be considered; these combine parametric models for representing series data and non-parametric models for including possible trends and seasonal effects, such as smoothing methods [33] and generalized additive models [34]. Although these regression methods take into account trends and seasonal components, the major disadvantage of using these techniques for fallen stock data is that the models did not consider the temporal correlation structure.

Similar drawbacks exist for methods related to statistical control process, which are mainly based on control charts (cumulative sum and exponentially weighted moving average) [35, 36], temporal scan statistics [37] or intervened times [38]. As in the case for regression techniques, these models seemed to be simpler in terms of interpretation and computation, but they did not adequately handle the autocorrelation structures of our data.

Other possible alternatives might be those based on models which take into account spatial information *(*i.e. cumulative sum charts, temporal scan statistics or spatial regression) [39–43] or methods of multivariate outbreak detection that dealt with several correlated series related to the same process [44, 45]. In contrast to previous techniques, spatial and multivariate models allow for the incorporation of correlation structures in the data, but there was the inconvenience that they would likely require the inclusion of new data, such as point locations of farms [39–43], or the application of dimension reduction methods [46].

### Comparison of baselines among different populations obtained from the models

Based on the selected ARIMA models, we observed that in region R2 all counties exhibited an increasing linear trend, and an annual or biannual seasonality. This region (and its provinces and counties) presented a more homogeneous behavior in mortality data than region R1.

This method could provide relevant information to point out or discard different health problems in these populations by comparing the number of mortality peaks detected among subpopulations, the time and season of detection, and the number of farms involved in each peak. For example, some mortality peaks detected at a regional level were also detected at a province and county levels (marked with dotted lines in Figs. 2 and 3). In particular, in R1, there were two peaks in weeks 466 and 470 that were detected at a region and county (C1, C2, and C3) levels. On the other hand, in R2, there was a mortality peak in week 469 that was identified at a region, province (P3), and county (C14) level. This suggested that the cattle populations of these hierarchical series were affected by a common cause. However, it is also important to underline that there were mortality peaks that were only detected at a county level, and were thus likely linked to the occurrence of more local events.

These results provide evidence that the analysis of bovine fallen stock data throughout several hierarchical administrative levels may bring relevant information to assess the evolution and the impact of different events occurred and/or detect earlier the start of a health threat that can spread to other areas. The assessment of mortality at lower aggregation level may put in evidence the occurrence of a local health problem (e.g. a food-borne infection locally transmitted), but also may sign the start of a contagious disease outbreak. In this last case, if mortality counts are uniquely studied at higher aggregation level, some problems may be unnoticed and get worse due to the lack of implementation of prevention measures on due time. Contrarily, the analyses conducted at higher aggregation level provide information to determine more easily the domain and global impact of a specific event on the study population and support the policy decision-making to coordinate actions of prevention and/or control at this level if it is necessary. Our outcomes (see Table 1 and Figs. 2 and 3) are consistent with the rationale that the analysis of this kind of data, aggregated at different hierarchical levels, enhances the detection of unusual aberrations, both globally and locally.

### Limitations of the study

Despite the benefits of our approach for AHSyS use, it is also important to discuss some constraints. First, the use of counts of fallen cattle as a proxy measure of mortality without taking into account the farm size could cause an over-expression of larger farms, masking unusual mortality events occurred in small farms. Besides, the baselines of fallen bovines might vary according to other factors, such as age, breed or sex. To enhance the accuracy of the system and identify unusual events of mortality for different populations, it would be important to include these covariates in the future. Unfortunately, these covariates could not be incorporated within our present study due to the lack of accuracy of the available records relating to these fields.

Second, it is important to recall that ARIMA models can only be used when populations and subpopulations show regular average counts. On this matter, it was first necessary to disaggregate and describe the series using HTS, to select those that could adequately be fitted using ARIMA models. Thus, the current results only covered a part of the subpopulations at province and county levels. A future innovation of the system to model the mortality in these subpopulations with very low counts would be to use integer-valued autoregressive models (INAR) [22], Hermite integer-valued autoregressive models (HINAR) [12], Bayesian approaches for seasonal count time series [20] or one of the other techniques reviewed in [25].

Third, the use of historical data without any prior exploration or filtering as a training set presented important limitations. For instance, if the population under study had suffered any unusual health problem during this period, the problem could be misclassified as a regular event and be included as a part of the basal pattern of the series, leading to a loss of sensitivity for similar alerts.

Fourth, to illustrate how our system forecasts and detects unusual aberrations, we used a conventional upper confidence limit of 95% a year and a half ahead. However, considering the unspecific nature of fallen stock data and the heterogeneity among subpopulations, it is plausible to think that the causes that underlie each mortality pattern and produced unusual aberrations could differ for each subpopulation. This implies that the optimal upper limits may have to be set according to the context of each subpopulation. Unfortunately, currently, the available information to determine these limits is scarce and unspecific, and any validation of this system in the field would require significant additional effort. Accordingly, we believe that the validation of this method as an early warning system would require more robust and updated data as well as the establishment of a more effective hierarchical communication between the field and each administrative level. All these aspects have led us to plan for a follow-on study to conduct an accurate validation using a multidisciplinary approach and more recent data.

## Conclusions

This work proposes a novel methodology to enhance the monitoring of bovine fallen stock data at different administrative levels and detect unusual mortality events. The system may provide essential information to identify spatiotemporal populations at high risk and allocate more effectively resources destined to control and prevent potential health problems. Moreover, the methodology proposed in this work can be applied to other populations and adapted to other AHSyS initiatives that require the follow-up of many populations with disparate husbandry systems.

## Methods

The populations under surveillance were dairy cattle in two Spanish regions: R1 (Principado de Asturias) and R2 (Catalonia). The region R1 included a large number of farms with small herd sizes, mostly in extensive husbandry systems. This region R1 had green landscapes and was characterized by coastal oceanic climate with moderate to high precipitations and moderate temperatures. In contrast, the region R2 had fewer farms but with larger herd sizes, most of which were in intensive production. Region R2 was characterized by a complex and diverse orography and a combination of a continental, coastal Mediterranean and alpine climates. These regions were monitored between 1st of Jan-2006 and 31th of Jun-2015.

During this period in both regions, and according to the European Union regulations [47], fallen animals were removed from farms using specialist disposal services. According to the Royal Decree 728/2007 (Annex III) [48], all the cattle farmers were obliged to use national fallen stock companies to collect and dispose their fallen stock and communicate the cattle casualties to the official animal health services.

### Types and sources of data

Two types of data from dairy cattle populations were used: fallen stock and demographic data. Fallen bovines data were provided by two national insurance companies: Entidad de Seguros Agrarios (ENESA) and Agrupación Española de Entidades Aseguradoras de los Seguros Agrarios Combinados S.A. (AGROSEGURO). For every visit where carcass disposal occurred, the following information was recorded: the identification code of the farm, date of pick-up, number of carcasses, and number of kilograms collected. On the other hand, demographic data yearly updated, were provided by the Sub-Directorate General for Animal Health and Hygiene and Traceability of the Ministry of Agriculture, Fisheries and Food of Spain (MAPA). This data set included: the identification code of the farm, type of production, number of dairy cattle per holding, and administrative aggregations (i.e. region, province, and county). Both sets of data, fallen bovine stock and bovine population, were merged in a final dataset using the identification code of the farm as a primary key.

According to the legal basis on the protection of personal data [49], the non-public staff and scientists involved in the analyses of these data signed a confidentiality agreement to set out the terms and conditions to limit the collection, handling, and disclosure of non-public information from farmers.

### Statistical analysis

We built a routine that consisted of a sequence of analytical methods designed to:

1. describe and select longitudinal fallen stock data aggregated at different levels (i.e., region, province, and county);
2. fit, for each subpopulation, appropriate time series models;
3. forecast the number of fallen bovine stock at n- ahead period; and
4. detect and register aberrations in mortality counts over time, considering the conventional upper confidence limit of 95%.

### Hierarchical time series structures

Firstly, for exploring and comparing baseline patterns from different sub-populations, fallen stock data aggregated at each of the three administrative levels were described and represented using HTS [13, 14]. HTS provide a rapid means to visualize, aggregate, and disaggregate series from different subpopulations. The HTS were designed by combining the information on the fallen dairy cattle at the most disaggregated level of the time series (in our case, counties) and the hierarchical organization. This dictates how the information, at the county level, had to be aggregated into provinces and regions. An explanatory illustration has been included in the supplemental material (S1 Fig) to explain the methodology used to build the HTS structures. The ‘base’ and ‘hts’ packages in the software R were used to support their creation [14].

### ARIMA modelling including trend and seasonal terms

The patterns and forecasting of weekly fallen bovines at different administrative levels were analyzed using ARIMA models that included trend and seasonal components as covariates. It is important to remark that ARIMA models are preferable for regular time series of normally distributed observations. Therefore, once the data were disaggregated into regions, provinces, and counties through HTS, each series that showed counts greater than or equal to 10 in average and normally distributed [50] were considered for ARIMA modelling. Since we were dealing with counts, most of the time series were Poisson distributed [12]. According to [51], for those series with an average higher than 10 counts, the Poisson distribution is well approximated by the Normal distribution; thus our selected series could be properly modelled through a classical parametric approach such ARIMA. These series also coincided with those administrative levels in which the dairy populations were larger within these two regions of study.

The ARIMA model, an extension of the ARMA (Autoregressive and Moving Average) model, has been widely used in the classical time series analysis and has also been applied in many contexts related to veterinary and public health [7, 50, 52, 53]. The autoregressive part of an ARIMA model (AR) indicates that the temporal response variable (i.e., weekly fallen bovine counts) is regressed by its lags. While the moving average part (MA) indicates that the model error is regressed by its lags (i.e., at time t, the lags of a random variable Z_t_ are essentially temporal delays such as Z_t − 1_, Z_t − 2_ …). The latter means that the error does not behave like white noise. These ARIMA models can also be used even if the series are non-stationary (i.e., when they present positive, negative, or quadratic trends, and/or annual and biannual seasonality, among other). In these cases, some initial differentiation can be applied one (d = 1) or more times (d > 1) to make the series stationary. Conceptually, when series are differentiating, the count at time t-1 is subtracting from the count at time t. The general ARIMA model is defined by the parameters p (autoregressive part), d (differentiation), and q (moving average part).

Let the random variable X_t_ be an ARMA (p, q) model such that:

1 $$ {\mathrm{X}}_{\mathrm{t}}=\upalpha +{\uprho}_1{\mathrm{X}}_{\mathrm{t}-1}+{\uprho}_2{\mathrm{X}}_{\mathrm{t}-2}+\dots +{\uprho}_{\mathrm{p}}{\mathrm{X}}_{\mathrm{t}-\mathrm{p}}+{\mathrm{Z}}_{\mathrm{t}}+{\uptheta}_1{\mathrm{Z}}_{\mathrm{t}-1}+{\uptheta}_2{\mathrm{Z}}_{\mathrm{t}-2}+\dots +{\uptheta}_{\mathrm{q}}{\mathrm{Z}}_{\mathrm{t}-\mathrm{q}} $$

where X_t_ is a stationary series at time t, α is the intercept of the model, ρ_1_, ρ_2_, …, ρ_p_ are the coefficients of the autoregressive term, θ_1_, θ_2_, …, θ_q_ are the coefficients of the moving average term, and Z_t_, Z_t − 1_, …, Z_t − q_ are the error terms of the model which are normally distributed. In case of lack of stationarity, the series X_t_ can be differentiated (d > 0) before estimating the parameters in Eq.1 to avoid possible trend and seasonal effects, leading to an ARIMA (p, d, q) model. Additionally, trends and seasonal influences can be included in the ARIMA (p, d, q) model as covariates. In particular, we accounted for trend and seasonality effects in the ARIMA (p, d, q) model by using the following equation:

2 $$ {\mathrm{Y}}_{\mathrm{t}}={\upgamma}_0+{\upgamma}_1\mathrm{t}+{\upgamma}_2\sin \left(\frac{2\uppi \mathrm{t}}{52}\right)+{\upgamma}_3\cos \left(\frac{2\uppi \mathrm{t}}{52}\right)+{\upgamma}_4\sin \left(\frac{2\uppi \mathrm{t}}{26}\right)+{\upgamma}_5\cos \left(\frac{2\uppi \mathrm{t}}{26}\right)+{\mathrm{X}}_{\mathrm{t}}, $$

where Y_t_ is the observed series at time t (which can be non-stationary), X_t_ is the stationary ARIMA (p, d, q) model expressed in Eq. 1., γ_1_is the trend coefficient, γ_2_ and γ_3_ are the coefficients of the annual seasonality aggregated at a weekly level, and γ_4_ and γ_5_ the coefficients of the biannual seasonality aggregated at a weekly level. Here the trigonometric part corresponds to the first and second-order Fourier terms commonly used in the analysis of time series [54].

These models were used to monitor the number of weekly fallen bovines for each subpopulation at every selected administrative level and identify abnormal peaks of mortality by comparing the predictions to the observations.

The number of weekly fallen bovines recorded between Jan-2006 and Jun-2015 at the regional, provincial, and county levels were divided into training and testing data sets. The data collected between Jan-2006 and Dec-2013 were used for estimating the model parameters for each population, while the data collected between Jan-2014 and Jun-2015 were used for testing the models and identifying unusual increases of mortality.

To estimate the most appropriate values of parameters p, d and q for the ARIMA models (Eq. 1), and also the linear trend and seasonal coefficients (Eq. 2) we built a programming routine. This routine explored a range of values between 0 and 5 for parameters p and q. For instance, if *p* = 5, the observed count at week t was regressed by the counts at weeks t-1, t-2, t-3, t-4 and t-5; while if q = 5, the error at week t was regressed by the errors at weeks t-1, t-2, t-3, t-4 and t-5. Here, the parameter d was constrained to a binary indicator, taking 0 (not differentiating) or 1 (differentiating once and removing the linear trend). Note that in those series in which the trend was not linear, but quadratic, the series was differentiated, and a coefficient of the linear trend (Eq. 2) was also included in the candidate model. The inclusion of this coefficient was because the trend was not linear, and hence it was not entirely removed by differentiating only once. In some scenarios, this problem could have been addressed by considering values of d greater than 1.

In order to select the most suitable ARIMA model for each time series among all candidates, three criteria were considered. The first one was based on the Bayesian Information Criterion (BIC) proposed by Schwarz [55]. The model with the smallest BIC was preferred over the whole set of candidates. The BIC was used because it typically provides more parsimonious models (in terms of the number of parameters) than those proposed by the Akaike Information Criterion (AIC). In addition, the BIC is also more robust than the AIC when coping with heterogeneity in samples [56]. The second and third criteria consisted of assessing the statistical significance of the model parameters given a significance level (i.e., 5%), and checking the behavior of the model residuals through the Auto-Correlation Function (ACF) and the Partial Auto-Correlation Function (PACF), respectively. The best ARIMA model was the one for which the third criterion was not only completely satisfied but also demonstrated good results for both the first and the second criteria. Full details can be found in [20, 21, 54].

Subsequently, each selected model was used to predict the expected number of carcasses that would be collected weekly between Jan-2014 and Jun-2015 in each population from every selected administrative aggregation. Those observed counts of fallen dairy cattle that exceeded the 95% confidence limits predicted by the selected ARIMA models were identified as mortality peaks. Reasonably, any relevant increase in mortality could sign an unusual event in the population, and thus require further investigation at field level to determine the specific causes. In case a presumed mortality peak would be detected, this routine provided a list with the identification codes of all involved farms. Additionally, the list also provided information on the number of carcasses recorded during the peak and over the preceding two weeks in those farms.

The programming routine was built using the base package of R [28].

## Supplementary information

**Additional file 1: S1 Fig** Illustration of hierarchical time series structures (hts).

**Additional file 2: S2 and S3 Figs** show the hierarchical time series by region, provinces and county in both regions R1 and R2. These Figures are computed by using the hts package included in R.

**Additional file 3: S1 Table**. Estimates and standard errors of the selected ARIMA (p,d,q) models for region R1, its province and counties between 2006 and 2013. **S2 Table**. Estimates and standard errors of the selected ARIMA(p,d,q) models for region R2, its provinces and counties between 2006 and 2013.

## Data Availability

The authors (AFF & AA) were required to analyze these data. Public access to these data is only possible through written permission of the Spanish Ministry of Agriculture and Fisheries and Food The access of programming routines and analyses generated during this study are possible through the contact of the corresponding author of this paper.

## Abbreviations

ACF: Auto-Correlation Function
AGROSEGURO: Agrupación Española de Entidades Aseguradoras de los Seguros Agrarios Combinados S.A
AIC: Akaike Information Criterion
AHSyS: Animal Health Syndromic Surveillance
AR: Autoregressive
ARIMA: Autoregressive Integrated and Moving Average
ARMA: Autoregressive and Moving Average
BIC: Bayesian Information Criterion
C: County
d: Order of differentiation
ENESA: Entidad de Seguros Agrarios
HINAR: Hermite Integer-Valued Autoregressive
HMM: Hidden Markov Models
HTS: Hierarchical time series structures
INAR: Integer-valued Autoregressive
MA: Moving Average
MAPA: Ministry of Agriculture, Fisheries and Food
p: Order of autoregressive part
P: province
PACF: Partial Auto-Correlation Function
q: Order of moving average part
R: Region
