# Supplementary material for: Enhancing the monitoring of fallen stock at different hierarchical administrative levels: an illustration on dairy cattle from regions with distinct husbandry, demographical and climate traits
Source: BMC Vet Res. 2020 Apr 14;16:110. doi: 10.1186/s12917-020-02312-8 (PMC7158015; doi:10.1186/s12917-020-02312-8)
Supplement: Supplementary file 1 — Additional file 1: S1 Fig Illustration of hierarchical time series structures (hts). [file 12917_2020_2312_MOESM1_ESM.docx]

Supporting Information

**S1 Fig Illustration of hierarchical time series structures (hts)**

$Y_{t} :$ observed series at time t at each administrative level

$Y_{x,t} :$ observations of series X at time t

$B_{t} :$ vector of all series at bottomlevel in time t

$S$: binary matrix indicating whether a given administrative level corresponds (1) or not (0) to higher administrative aggregations, and so that should be conveniently aggregated to those higher levels

$Y_{t}=\left( \begin{aligned} Y_{R1=P1,t} \\ Y_{C1,t} \\ Y_{C2,t} \\ Y_{C3,t} \\ Y_{C4,t} \\ Y_{C5,t} \\ Y_{C6,t} \\ Y_{C7,t} \end{aligned} \right)=\left( \begin{aligned} \begin{matrix} 1 \\ 1 \\ 0 \end{matrix} \\ 0 \\ 0 \\ 0 \\ 0 \\ 0 \\ 0 \end{aligned}\begin{aligned} \begin{matrix} 1 \\ 0 \\ 1 \end{matrix} \\ 0 \\ 0 \\ 0 \\ 0 \\ 0 \\ 0 \end{aligned}\begin{aligned} \begin{matrix} 1 \\ 0 \\ 0 \end{matrix} \\ 1 \\ 0 \\ 0 \\ 0 \\ 0 \\ 0 \end{aligned}\begin{aligned} \begin{matrix} 1 \\ 0 \\ 0 \end{matrix} \\ 0 \\ 1 \\ 0 \\ 0 \\ 0 \\ 0 \end{aligned}\begin{aligned} \begin{matrix} 1 \\ 0 \\ 0 \end{matrix} \\ 0 \\ 0 \\ 1 \\ 0 \\ 0 \\ 0 \end{aligned}\begin{aligned} \begin{matrix} 1 \\ 0 \\ 0 \end{matrix} \\ 0 \\ 0 \\ 0 \\ 1 \\ 0 \\ 0 \end{aligned}\begin{aligned} \begin{matrix} 1 \\ 0 \\ 0 \end{matrix} \\ 0 \\ 0 \\ 0 \\ 0 \\ 1 \\ 0 \end{aligned}\begin{aligned} \begin{matrix} 1 \\ 0 \\ 0 \end{matrix} \\ 0 \\ 0 \\ 0 \\ 0 \\ 0 \\ 1 \end{aligned} \right)\left( \begin{aligned} \begin{matrix} Y_{C1R1,t} \\ Y_{C2R1,t} \\ Y_{C3R1,t} \end{matrix} \\ Y_{C4R1,t} \\ Y_{C5R1,t} \\ Y_{C6R1,t} \\ Y_{C7R1,t} \end{aligned} \right)$

Y_t_ = SB_t_

B_t_

S

$Y_{t}=\left( \begin{aligned} Y_{R2,t} \\ Y_{P2,t} \\ Y_{P3,t} \\ Y_{C8P2,t} \\ Y_{C9P2,t} \\ Y_{C10P2,t} \\ Y_{C11P2,t} \\ Y_{C12P3,t} \\ Y_{C13P3,t} \\ Y_{C14P3,t} \end{aligned} \right)=\left( \begin{aligned} 1 \\ 1 \\ 0 \\ 1 \\ 0 \\ 0 \\ 0 \\ 0 \\ 0 \\ 0 \end{aligned}\begin{aligned} 1 \\ 1 \\ 0 \\ 0 \\ 1 \\ 0 \\ 0 \\ 0 \\ 0 \\ 0 \end{aligned}\begin{aligned} 1 \\ 1 \\ 0 \\ 0 \\ 0 \\ 1 \\ 0 \\ 0 \\ 0 \\ 0 \end{aligned}\begin{aligned} 1 \\ 1 \\ 0 \\ 0 \\ 0 \\ 0 \\ 1 \\ 0 \\ 0 \\ 0 \end{aligned}\begin{aligned} 1 \\ 0 \\ 1 \\ 0 \\ 0 \\ 0 \\ 0 \\ 1 \\ 0 \\ 0 \end{aligned}\begin{aligned} 1 \\ 0 \\ 1 \\ 0 \\ 0 \\ 0 \\ 0 \\ 0 \\ 1 \\ 0 \end{aligned}\begin{aligned} 1 \\ 0 \\ 1 \\ 0 \\ 0 \\ 0 \\ 0 \\ 0 \\ 0 \\ 1 \end{aligned} \right)\left( \begin{aligned} \begin{matrix} Y_{C8P2,t} \\ Y_{C9P2,t} \\ Y_{C10P2,t} \end{matrix} \\ Y_{C11P2,t} \\ Y_{C12P3,t} \\ Y_{C13P3,t} \\ Y_{C14P3,t} \end{aligned} \right)$

Y_t_ = SB_t_

B_t_

S
