# Supplementary material for: Enhancing the monitoring of fallen stock at different hierarchical administrative levels: an illustration on dairy cattle from regions with distinct husbandry, demographical and climate traits
Source: BMC Vet Res. 2020 Apr 14;16:110. doi: 10.1186/s12917-020-02312-8 (PMC7158015; doi:10.1186/s12917-020-02312-8)
Supplement: Supplementary file 2 — Additional file 2: S2 and S3 Figs show the hierarchical time series by region, provinces and county in both regions R1 and R2. These Figures are computed by using the hts package included in R. [file 12917_2020_2312_MOESM2_ESM.docx]

S2 and S3 Figs show the hierarchical time series by region, provinces and county in both regions R1 and R2. These Figures are computed by using the hts package included in R.


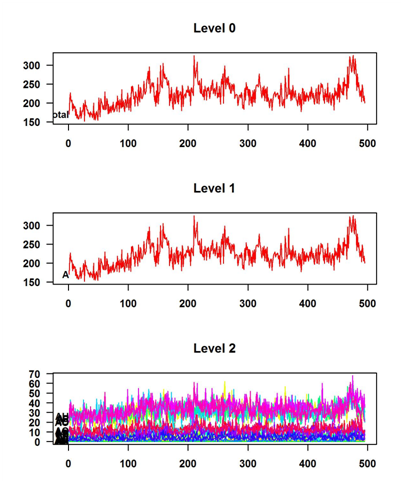


SFig1. Hierarchical time series by region (Level 0), provinces (Level 1) and county (Level 2) in region R1.

* Note that the different colors at Level 2 represent each of the counties that can be aggregated at a province (Level 1) and region (Level 0) levels.


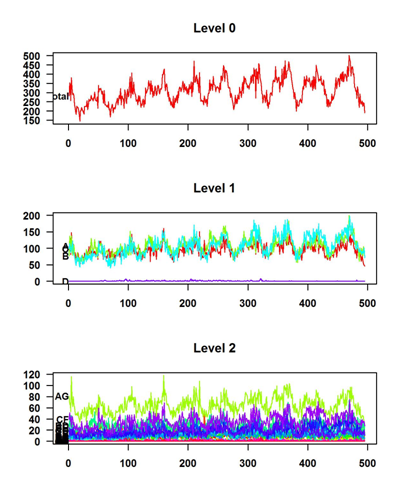


Sfig2. Hierarchical time series by region (Level 0), provinces (Level 1) and county (Level 2) in region R2.
* Note that the different colors at Level 2 represent each of the counties that can be aggregated at a province (Level 1) and region (Level 0) levels.
