# Supplementary material for: Enhancing the monitoring of fallen stock at different hierarchical administrative levels: an illustration on dairy cattle from regions with distinct husbandry, demographical and climate traits
Source: BMC Vet Res. 2020 Apr 14;16:110. doi: 10.1186/s12917-020-02312-8 (PMC7158015; doi:10.1186/s12917-020-02312-8)
Supplement: Supplementary file 3 — Additional file 3: S1 Table. Estimates and standard errors of the selected ARIMA (p,d,q) models for region R1, its province and counties between 2006 and 2013. S2 Table. Estimates and standard errors of the selected ARIMA(p,d,q) models for region R2, its provinces and counties between 2006 and 2013. [file 12917_2020_2312_MOESM3_ESM.docx]

**S1 Table. Estimates and standard errors of the selected ARIMA (p,d,q) models for region R1, its province and counties between 2006 and 2013.**

| **Zones of study** | **ARIMA(p, d, q)** | **Estimated equation of the model** |
| --- | --- | --- |
| R1-P1 | 1,0,1 | $X_{t}=196.841+0.086t+4.676\sin\left( \frac{2\pi t}{52} \right)-8.238\cos\left( \frac{2\pi t}{52} \right)-9.907\sin\left( \frac{2\pi t}{26} \right)+Y_{t}$  s.e. $14.513 0.057 2.480 2.431 1.639$  $(1-0.980L)Y_{t}=Z_{t}-0.809Z_{t-1}$  s.e. $0.012$ $0.040$ |
| C1 | 0,1,1 | $X_{t}=0.007t+1.739\cos\left( \frac{2\pi t}{52} \right)+1.741\sin\left( \frac{2\pi t}{26} \right)+ Y_{t}$  s.e. $0.019 0.471 0.435$  $(1-L)Y_{t}=Z_{t}-0.941Z_{t-1}$  s.e. $0.016$ |
| C2 | 0,1,1 | $X_{t}=1.207\cos\left( \frac{2\pi t}{52} \right)+1.354\sin\left( \frac{2\pi t}{26} \right)+ Y_{t}$  s.e. $0.519 0.472$  $(1-L)Y_{t}=Z_{t}-0.934Z_{t-1}$  s.e. $0.022$ |
| C3 | 1,0,1 | $X_{t}=27.985+0.009t+1.722\sin\left( \frac{2\pi t}{26} \right)+ Y_{t}$  s.e. $0.979 0.004 0.511$  $(1-0.844L)Y_{t}=Z_{t}-0.732Z_{t-1}$  s.e. $0.086$ $0.109$ |
| C4 | 0,1,1 | $X_{t}=0.022t+1.062\sin\left( \frac{2\pi t}{26} \right)+ Y_{t}$  s.e. $0.019 0.492$  $(1-L)Y_{t}=Z_{t}-0.949Z_{t-1}$  s.e. $0.014$ |
| C5 | 1,0,1 | $X_{t}=10.875+0.004t+0.618\cos\left( \frac{2\pi t}{52} \right)+Y_{t}$  s.e. $0.804 0.003 0.317$  $(1-0.964L)Y_{t}=Z_{t}-0.920Z_{t-1}$  s.e. $0.031$ $0.044$ |
| C6 | 0,1,1 | $X_{t}=0.125t+2.414\cos\left( \frac{2\pi t}{52} \right)+1.213\sin\left( \frac{2\pi t}{26} \right)+1.061\cos\left( \frac{2\pi t}{26} \right)+ Y_{t}$  s.e. $0.023 0.523 0.477 0.474$  $(1-L)Y_{t}=Z_{t}-0.935Z_{t-1}$  s.e. $0.018$ |
| C7 | 1,0,1 | $X_{t}=10.083-0.006t+0.616\sin\left( \frac{2\pi t}{52} \right)+ Y_{t}$  s.e. $1.242 0.005 0.302$  $(1-0.979L)Y_{t}=Z_{t}-0.909Z_{t-1}$  s.e. $0.014$ $0.029$ |

**S2 Table. Estimates and standard errors of the selected ARIMA(p,d,q) models for region R2, its provinces and counties between 2006 and 2013.**

| **Zones of study** | **ARIMA(p, d, q)** | **Estimated equation of the model** |
| --- | --- | --- |
| R2 | 0,1,1 | $X_{t}=0.229t-23.444\sin\left( \frac{2\pi t}{52} \right)+44.591\cos\left( \frac{2\pi t}{52} \right)+31.505\sin\left( \frac{2\pi t}{26} \right)+14.069\cos\left( \frac{2\pi t}{26} \right)+Y_{t}$  s.e. $0.226 3.363 3.294 2.423 2.400$  $(1-L)Y_{t}=Z_{t}-0.855Z_{t-1}$  s.e. $0.032$ |
| P2 | 0,1,1 | $X_{t}=0.110t-11.840\sin\left( \frac{2\pi t}{52} \right)+17.930\cos\left( \frac{2\pi t}{52} \right)+11.599\sin\left( \frac{2\pi t}{26} \right)+6.642\cos\left( \frac{2\pi t}{26} \right)+ Y_{t}$  s.e. $0.096 1.558 1.525 1.201 1.190$  $(1-L)Y_{t}=Z_{t}-0.882Z_{t-1}$  s.e. $0.032$ |
| P3 | 1,0,1 | $X_{t}=89.606+0.079t-7.553\sin\left( \frac{2\pi t}{52} \right)+13.873\cos\left( \frac{2\pi t}{52} \right)+11.596\sin\left( \frac{2\pi t}{26} \right)+4.710\cos\left( \frac{2\pi t}{26} \right)+ Y_{t}$  s.e. $4.054 0.017 1.855 1.817 1.292 1.280$  $(1-0.922L)Y_{t}=Z_{t}-0.762Z_{t-1}$  s.e. $0.035$ $0.059$ |
| C8 | 0,1,1 | $X_{t}=0.037t-1.891\sin\left( \frac{2\pi t}{52} \right)+2.040\cos\left( \frac{2\pi t}{52} \right)+1.385\sin\left( \frac{2\pi t}{26} \right)+0.862\cos\left( \frac{2\pi t}{26} \right)+ Y_{t}$  s.e. $0.018 0.442 0.433 0.401 0.398$  $(1-L)Y_{t}=Z_{t}-0.941Z_{t-1}$  s.e. $0.029$ |
| C9 | 2,1,2 | $X_{t}=0.031t-3.155\sin\left( \frac{2\pi t}{52} \right)+5.098\cos\left( \frac{2\pi t}{52} \right)+2.661\sin\left( \frac{2\pi t}{26} \right)+ Y_{t}$  s.e. $0.013 0.788 0.774 0.622$  $(1-0.642L-0.149L^{2})(1-L)Y_{t}=Z_{t}-0.855Z_{t-1}-0.594Z_{t-2}$  s.e. $0.133$ $0.058 0.128 0.124$ |
| C10 | 0,1,1 | $X_{t}=0.040t-3.375\sin\left( \frac{2\pi t}{52} \right)+5.644\cos\left( \frac{2\pi t}{52} \right)+4.315\sin\left( \frac{2\pi t}{26} \right)+3.270\cos\left( \frac{2\pi t}{26} \right)+ Y_{t}$  s.e. $0.022 0.650 0.639 0.605 0.602$  $(1-L)Y_{t}=Z_{t}-0.951Z_{t-1}$  s.e. $0.023$ |
| C11 | 4,1,2 | $X_{t}=0.012t-3.890\sin\left( \frac{2\pi t}{52} \right)+4.111\cos\left( \frac{2\pi t}{52} \right)+1.988\sin\left( \frac{2\pi t}{26} \right)+1.629\cos\left( \frac{2\pi t}{26} \right)+ Y_{t}$  s.e. $0.009 0.885 0.863 0.550 0.544$  $(1-0.718L+0.277L^{2}+0.244L^{3}-0.155L^{4})(1-L)Y_{t}=Z_{t}-1.617Z_{t-1}+0.617Z_{t-2}$  s.e. $0.147$ $0.059 0.081 0.055 0.145 0.144$ |
| C12 | 1,0,1 | $X_{t}=13.561+0.009t-1.093\sin\left( \frac{2\pi t}{52} \right)+3.029\cos\left( \frac{2\pi t}{52} \right)+2.300\sin\left( \frac{2\pi t}{26} \right)+1.208\cos\left( \frac{2\pi t}{26} \right)+ Y_{t}$  s.e. $0,970 0,004 0,479 0,471 0,377 0,374$  $\left( 1-0.922L \right)Y_{t}=Z_{t}-0.833Z_{t-1}$  s.e. $0.043$ $0.060$ |
| C13 | 0,1,1 | $X_{t}=0.021t-2.697\sin\left( \frac{2\pi t}{52} \right)+2.700\cos\left( \frac{2\pi t}{52} \right)+3.056\sin\left( \frac{2\pi t}{26} \right)+1.386\cos\left( \frac{2\pi t}{26} \right)+ Y_{t}$  s.e. $0.020 0.517 0.508 0.471 0.468$  $(1-L)Y_{t}=Z_{t}-0.941Z_{t-1}$  s.e. $0.022$ |
| C14 | 3,1,1 | $X_{t}=0.011t-1.522\sin\left( \frac{2\pi t}{52} \right)+2.700\cos\left( \frac{2\pi t}{52} \right)+4.800\sin\left( \frac{2\pi t}{26} \right)+1.401\cos\left( \frac{2\pi t}{26} \right)+ Y_{t}$  s.e. $0.005 0.820 0.811 0.732 0.727$  $(1-0.093L-0.146L^{2}-0.185L^{3})(1-L)Y_{t}=Z_{t}-1.000Z_{t-1}$  s.e. $0.049$ $0.049 0.049 0.007$ |

The operator L means $LY_{t}=Y_{t-1}$ and $L^{k}Y_{t}=Y_{t-k}$.
